# A Comparative Evaluation of Mechanical Properties of Four Different Restorative Materials: An *In Vitro* Study

**DOI:** 10.5005/jp-journals-10005-1592

**Published:** 2019

**Authors:** Nahid Iftikhar, Devashish, Binita Srivastava, Nidhi Gupta, Natasha Ghambir, Rashi-Singh

**Affiliations:** 1,3–6 Department of Pedodontics and Preventive Dentistry, Santosh Dental College and Hospital, Ghaziabad, Uttar Pradesh, India; 2 Department of Pedodontics and Preventive Dentistry, Army Dental Corp, India

**Keywords:** Compressive strength, Diametral tensile strength, Restorative materials

## Abstract

**Objectives:**

The purpose of this study is to compare the mechanical properties (compressive strength (CS) and diametral tensile strength (DTS)) of four different restorative materials: conventional glass ionomer (Fuji IX), ClearFil AP-X, Filtex Z350-XT, and Cention N.

**Materials and methods:**

Specimens (*n* = 80) were prepared from Fuji IX, ClearFil AP-X, Filtex Z350-XT, and Cention N for testing compressive strength and DTS.

**Statistical analysis:**

Results obtained were subjected to one-way ANOVA and Tukey's *post hoc* test at significance (*p* < 0.001).

**Results:**

There were significant differences among restorative materials tested. ClearFil AP-X exhibits the highest mechanical properties (CS and DTS) and least values were obtained by the Fuji IX.

**Conclusion:**

Strength is one of the most important criteria for the selection of a restorative material. Stronger materials better resist deformation and fracture, presenting more equitable stress distribution, greater probability, and greater stability of clinical success.

**How to cite this article:**

Iftikhar N, Devashish, *et al.* A Comparative Evaluation of Mechanical Properties of Four Different Restorative Materials: An *In Vitro* Study. Int J Clin Pediatr Dent 2019;12(1):47–49.

## INTRODUCTION

Dental caries is an age-old disease which has been the blight of affliction in the oral cavity. It is one of the most widespread diseases in the population due to high ingestion of carbohydrates and lack of knowledge regarding proper oral hygiene methods. Once it occurs, restoring the carious lesions becomes compulsory. Hence, when the choice of restorative materials is made, certain properties should be considered, such as adhesion to the tooth structure, load-bearing strength of the materials, biocompatibility retention, and simplicity of application.^1^ For posterior restorations, the materials have to withstand forces of compression and tension. From the earlier materials like silver amalgam to latest advances in composites, posterior restorative materials have been constantly evolving. Each material used to restore posterior teeth has specific advantages and disadvantages and these should be suspiciously weighed before selecting a restorative material.^2^

Glass ionomer cement (GIC) was introduced in 1972 by Wilson and Kent for restorative and preventive applications. Its unique properties such as adhesion to moist teeth, lack of exothermic polymerization, anticarcinogenic character, excellent adhesion to dentin, satisfactory biocompatibility, and coefficient of thermal expansion similar to that of tooth make it an important material for dental restorations. However, one of the major drawbacks of GIC is its weak mechanical properties such as toughness, brittleness, and low compressive strength, because of which alternative filling materials have been researched.^3–5,13^

In the last four decades, there have been tremendous improvements and innovations in the development of more constant composite materials. These developments were focused mainly on reducing polymerization shrinkage and improving the mechanical properties. The progression in filler and polymer technology of dental composite resins has led to a wide range of composite material selections based on clinical situation.^6^ Nanotechnology has a great impact on restorative dentistry by offering refinements to the already available resin-based composite system. These materials were introduced in 2002 which were formulated with nanomer and nanocluster filler particles, which were expected to be useful for all restorative applications. But they have the limitation that they cannot be used as posterior restoration materials where isolation is poor and wear is high.^7^

Cention N (Ivoclar Vivadent) is a recently introduced tooth-colored, restorative filling material for bulk placement in retentive preparations with or without the application of an adhesive. It is an “alkasite” restorative which is a new category of filling material, like compomer, and is essentially a subgroup of the composite resin. Cention N is a urethane dimethacrylate (UDMA)-based, self-curing powder/liquid restorative with optional additional light curing. The liquid comprises of dimethacrylates and initiators, while the powder contains various glass fillers, initiators, and pigments. It is radio opaque and contains alkaline glass fillers capable of releasing fluoride, calcium, and hydroxide ions. Due to the sole use of cross-linking methacrylate monomers in combination with a stable, efficient self-cure initiator, Cention N displays a high polymer network density and degree of polymerization over the complete depth of the restoration.^8,9^

The success of dental treatment depends not only on biological, physical, chemical, and pathophysiological principles but also on the adequate and significant knowledge of the mechanical properties of dental tissues and materials.^10^

The purpose of this study was to investigate and evaluate the mechanical properties of different restorative materials.

## MATERIALS AND METHODS

A total of 80 specimens (*n* = 80) were prepared with the four materials used for the study (Table 1). A total of 40 specimens were used for testing compressive strength and the remaining 40 were used for the diametral tensile strength (DTS) testing. The specimens were prepared in the cylindrical molds with standard dimensions of the American Dental Association (ADA) specification.^12^ All the materials were mixed and prepared according to the instruction from the manufacturer. The specimens were made at room temperature 23 ± 2°C, with a relative air humidity of 50 ± 10%. The mixed material was slowly inserted in the molds and plates were placed above it followed by slight application of pressure for 20 seconds. The excess material was extruded from the top. The test specimens were subjected to a water bath at 37 ± 1°C for 1 hour before testing.

### Compressive Strength Testing

**Figs 1A and B:**
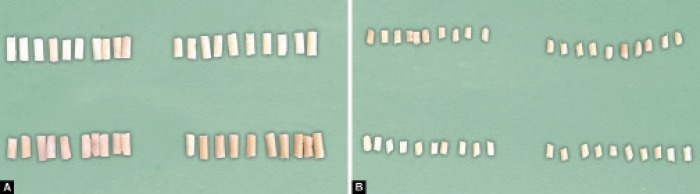
(A) Specimens for compressive strength; (B) Specimens for diametral tensile

**Fig. 2:**
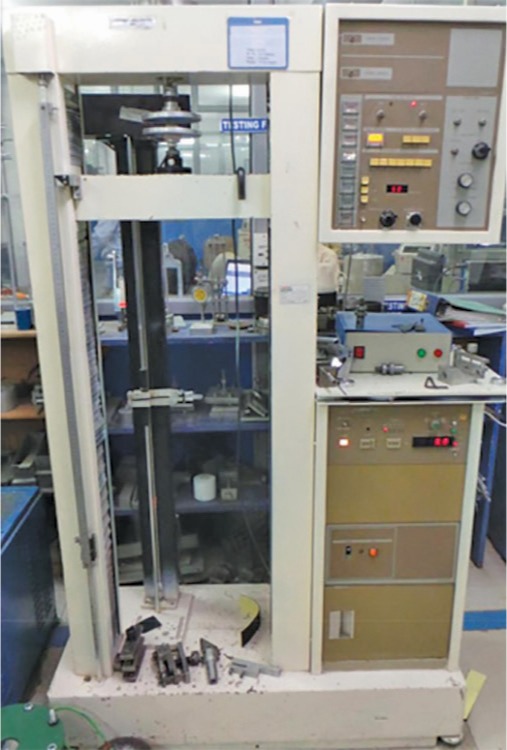
Instron universal testing machine

According to the ADA specification, cylindrical specimens were prepared in molds with dimensions of 6 mm in diameter and 12 mm in height (Fig. 1A). This test was carried out using the Instron universal testing machine that has a crosshead speed of 1.0 mm/minute (Fig. 2). Each sample was placed with the flat ends between the platens of the specimens. The maximum load applied to fracture the specimens was recorded and the compressive strength was calculated using the following formula: CS = 4*P*/*πD*^2^, where *P* is the maximum applied load (N) and *D* is the measured diameter of the sample (mm).

### DTS Testing

For the DTS test, the dimension of specimens was 6.0 mm in diameter and 3.0 mm in height (Fig. 1B). The sample was placed with the flat ends perpendicular to the platens in the Instron universal testing machine so that the load will be applied to the diameter of the specimens. When the maximum load was applied to the fracture, the specimens were recorded at a crosshead speed of 0.1 mm/minute and the DTS was calculated using the following formula: *T* = 2*P*/*πDL*, where *P* is the maximum applied load (N), *D* is the measured diameter of the sample (mm), and *L* is the measured length of the sample (mm).

**Table 1:** The restorative materials tested in this study

*Materials*	*Manufacturers*
GIC (Fuji IX)	GC Corp., Japan
ClearFil AP-X	Kuraray
Filtex Z350-XT	3M ESPE
Cention N	Ivoclar Vivadent

### Statistical Analysis

Statistical analysis of compressive strength and DTS testing was performed and the mean value with its standard deviation was calculated for each restorative material. Results were subjected to one-way ANOVA for comparison between groups and Tukey's *post hoc* test to compare the materials among groups. *p* < 0.0001 is obtained which indicates a highly statistically significant difference between tested materials.

## RESULTS

**Fig. 3:**
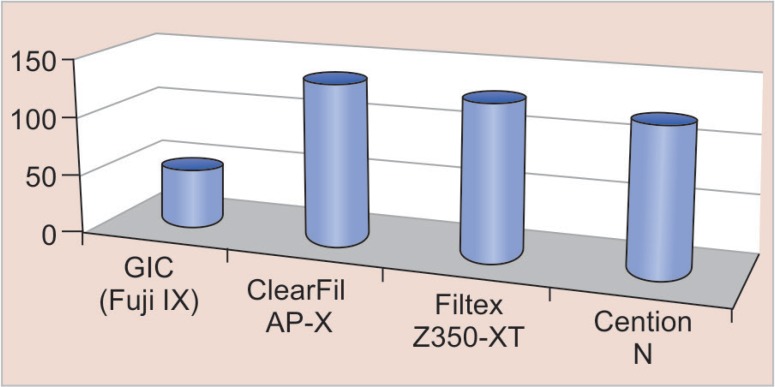
Compressive strength of restorative materials tested

**Fig. 4:**
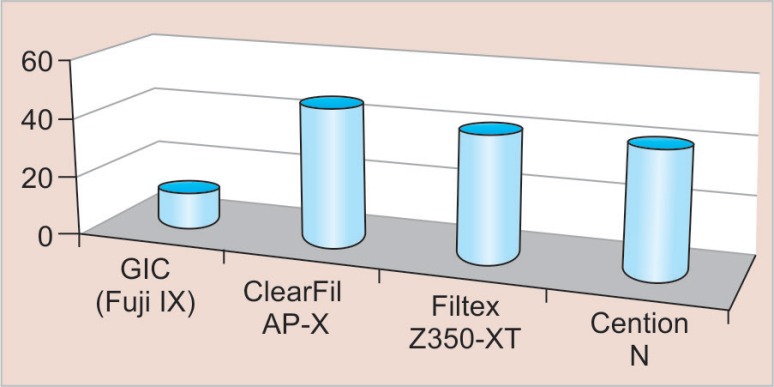
DTS of restorative materials tested

The compressive strength of GIC (Fuji IX), ClearFil AP-X, Filtex Z350-XT, and Cention N was 47 ± 10, 134 ± 26, 126 ± 19, and 121 ± 33 MPa, respectively (Fig. 3). The ClearFil AP-X had the highest strength. The GIC (Fuji IX) had a compressive strength significantly lower compared with Filtex Z350-XT and Cention N. Similarly, the DTS of the four materials is represented (Fig. 4). The DTS also showed a similar pattern with 11.8 ± 2.3 MPa for GIC (Fuji IX), which was significantly lower compared with ClearFil AP-X (46.4 ± 17.5), Filtex Z350-XT (42.3 ± 28.4), and Cention N (41.0 ± 12.5) MPa.

## DISCUSSION

Among all the restorative materials available, composite resin (ClearFil AP-X) has become the material of choice for restoration of all teeth. The recognition of resin-based composite restoration has increased because of its excellent esthetic and other favorable characteristics. In the present study, comparing the results obtained, the null hypothesis was rejected as there was a significant difference in mechanical properties (compressive strength, DTS) among the newer posterior restorative material tested. The restorative materials used in the oral environment are subjected to various occlusal forces.

In this study, the mechanical properties of dental hard tissues and various dental restorative materials were compared and studied with respect to the bite force. The analysis of the compressive strength and DTS is important for the comparison of mechanical properties of dental materials which reflect which material is better to perform clinically and is resistant to the masticatory forces.

The result of the study indicated that the four materials tested in the study differed statistically in terms of compressive strength and DTS with a *p* value of 0.001, which suggests a significant difference in mechanical properties. The findings of this work have shown that the nanofilled composite (ClearFil AP-X) has relatively high compressive strength (134.938 MPa). Basically, the compressive strength test evaluates the masticatory forces of restorative materials, especially posterior composites, diametral tensile was also high in ClearFil AP-X (46.449 MPa) as compared with the other tested materials. ClearFil AP-X showed a statistically significant difference in compressive strength and DTS with GIC (Fuji IX) (0.001) with the *p* value significant at 0.005. The value of compressive strength and diametral tensile in Filtex Z350-XT was 126.75 MPa and 42.308 MPa, respectively, where Cention N (121.395 MPa, 41.097 MPa) also showed good mechanical properties. The weakest mechanical properties were obtained by GIC (Fuji IX) with the mean value of 47.842 MPa in compressive strength and 11.800 MPa in DTS. There are various studies with regard to the comparative evaluation of mechanical properties of restorative materials showing dichotomy of results. This was in accordance with Kumar et al.^11^ who did a comparative study on mechanical properties of direct core build-up materials. They concluded that the composite had high mechanical properties and GICs showed the weakest; this is in agreement with our study.

## CONCLUSION

In the present study, it can be concluded that the mean compressive strength and DTS values of all the four restorative materials were significantly different because the composite materials available have a variation in composition and viscosity. The nanohybrid composite ClearFil AP-X has the highest compressive strength and DTS and the properties of Z350-XT and Cention N were almost similar. GIC (Fuji IX) exhibited the least values when compared with that of the other materials. In the present study, the null hypothesis was not accepted; further clinical studies should be carried out as all nanocomposites exhibited different mechanical properties.
